# The Effect of Oral Administration of Bisphenol A and AF on Their Deposition in the Body Organs of Growing Pigs and the Relationship to Growth Rate

**DOI:** 10.3390/ani15213214

**Published:** 2025-11-05

**Authors:** Ivan Bahelka, Roman Stupka, Kateřina Zadinová, Michal Šprysl, Jaroslav Čítek

**Affiliations:** Department of Animal Science, Faculty of Agrobiology, Food and Natural Resources, University of Life Sciences in Prague, Kamýcká 129, 165 00 Prague, Czech Republic; bahelka@af.czu.cz (I.B.); stupka@af.czu.cz (R.S.); sprysl@af.czu.cz (M.Š.); citek@af.czu.cz (J.Č.)

**Keywords:** bisphenol A, bisphenol AF, pig, deposition in tissue, growth

## Abstract

Bisphenol A (BPA) and its analogues, such as bisphenol AF (BPAF), are widely used mainly in the plastic industry. These compounds can leach into the environment and negatively impact living organisms, including farm animals such as pigs. The aim of the study was to evaluate concentrations of BPA and BPAF in plasma and tissues of pigs after oral administration at a lower—20 µg (10 µg BPA + 10 µg BPAF)/kg body weight (b.w.)/day—and higher dose—60 µg (30 µg BPA + 30 µg BPAF)/kg b.w./day—for 21 days. The concentrations of BPA and BPAF in plasma and liver were monitored immediately after finishing the bisphenols administration, as well as two and four weeks after that. Generally, pigs receiving higher doses of BPA/BPAF showed the highest levels across most parameters, followed by those with lower doses and the control group. Results showed that both bisphenols remained in the organs of experimental pigs for two and four weeks after the administration was completed, respectively. Results also showed that the growth rate did not have a significant effect on the accumulation of bisphenols in pig tissues. The results of the present study have shown that oral administration of bisphenols led to an increase in the concentration of BPA and BPAF in plasma and other tissues of young pigs. Moreover, the fact that both BPA and BPAF were also detected in control pigs suggests that the farm environment was contaminated with these substances. This may pose a risk not only to the health and performance of pigs but also to human health, through the consumption of pig organs.

## Simple Summary

Bisphenol A (BPA) and its analogue, bisphenol AF (BPAF), are artificial substances that are widely used in the modern plastic industry. It is well known that both have a significant negative impact on the health of experimental animals as well as humans. However, their incidence and deposition in the organs of farm animals are quite limited. In the present study, the effect of oral administration of BPA and BPAF on accumulation in some tissues and the relationship to the growth rate of piglets was evaluated. The results showed that both bisphenols are present in the plasma and organs of pigs not only immediately after slaughter but also at two and four weeks post-slaughter.

## 1. Introduction

Industrial manufacturing produces a wide range of substances that are beneficial to human civilization’s needs, but on the other hand, have an evidently harmful effect on the environment and living organisms, including humans. One of these substances is plastic materials. Modern plastic materials can contain various additional components that enhance their properties. Bisphenol A (BPA) is one of the chemicals in this group that is commonly produced and used worldwide. Since the 1940s, it has been used in the manufacturing of synthetic polymers, including epoxy resins and polycarbonates. These materials are used in many consumer products, such as bottles, toys, pacifiers, personal care products, dental sealants, eyeglass lenses, sports safety and medical equipment, water pipes, furniture, electronic devices and compact discs, dye developer in thermal paper, household goods, automobile parts, and food and beverage containers [1]. Due to its massive production and vast applications, BPA is present in the atmosphere and dust [2], soil and tap water [3], and foodstuffs and drinking water [4], as well as living organisms, including humans [5].

It is well known that BPA may enter the human body via the skin or respiratory system [6], but primarily through the ingestion of contaminated foodstuffs and drinks [7]. BPA is classified as an “endocrine disruptor” or “endocrine-disrupting compound” (EDC), pointing out its ability to damage the hormonal system of animals and humans. This detrimental impact is due to its similarity to estrogen; it binds to estrogen receptors and affects the activity of reproductive organs. However, BPA has been proven to impact not only reproductive functions but also other systems, such as the nervous, digestive, and immune systems [8]. BPA is also suspected to be a teratogen—it is present in the placenta and amniotic fluid, suggesting that it can penetrate the embryo during pregnancy and have a harmful effect on its development, even at very low doses [9]. Recently, numerous investigations have shown some associations between exposure to BPA and the risk of hypertension and heart diseases, obesity, diabetes, and cancer—especially breast and prostate cancer—in animals and humans [10,11]. These findings collectively suggest that BPA is considered a highly hazardous endocrine disruptor. For this reason, several countries have attempted to limit their production and introduce restrictions on the use of these products in certain applications, such as toys, baby bottles, cosmetics, and dental sealants. BPA is also gradually being replaced by its analogues. In 2023, the European Food Safety Authority (EFSA) significantly reduced the tolerable daily intake (TDI) of bisphenol A (BPA) to 0.2 nanograms (ng) per kilogram of body weight per day. As of January 2025, BPA has been banned in all materials that are intended to come into contact with food within the European Union [12,13].

One of these is bisphenol AF (BPAF—1,1,1,3,3,3—hexafluoro—2,2—bis (4-hydroxyphenyl) propane), which is extensively used in the production of fluoroelastomers and fluoropolymers as a rubber bridging substance, as well as for the production of plastic materials such as polyesters, polyamides, polycarbonates, and food-contact polymers [14]. Similarly to BPA, BPAF has been detected in various environmental matrices, including indoor dust, sewage sludge [15], soils, sediments, and surface waters [16], as well as in some foodstuffs and beverages [17]. In humans, BPAF has been found in plasma, urine, and breast milk [18]. Currently, the estimated dietary intake of BPAF is 0.49 ng/kg body weight/day for men and 0.50 for women [19]. Several studies have revealed that BPAF acts as an agonist for estrogen receptor (ER) α and as an antagonist of ER β [20]. BPAF also reduces the fertilization rate of offspring eggs and the survival rate, as well as steroidogenesis [21], cell cycle, nuclear morphology, and oocyte maturation, while increasing oxidative stress and DNA damage at concentrations as low as 1 ng/mL [22]. It can also disrupt the balance of hormones and cause other developmental and reproductive disorders, as well as affect the expression of genes involved in cardiovascular diseases, immune system function, and fetal development [23].

The aim of the study was to evaluate the effect of two different orally administered doses of bisphenol A and bisphenol AF on their concentrations and accumulation in plasma, as well as in selected internal organs and tissues of pigs, to assess the elimination of these compounds from the organism. This effect was monitored not only immediately after the administration of bisphenols was completed, but also two and four weeks afterward. Moreover, the study investigated the relationship between the growth rate of pigs and the deposition of bisphenols in their organs: that is, whether faster-growing pigs, which likely consume larger amounts of feed and therefore receive higher doses of bisphenols, had higher concentrations of these compounds in their organs compared to slower-growing ones.

## 2. Materials and Methods

### 2.1. Animals and Diet

Thirty-six weaned pigs (18 barrows and 18 gilts) at the age of 42 days were included in the present study. They originated from the commercial private swine farm located in the north-west part of the Czech Republic. After transport, pigs were housed and kept in standard stock-farming conditions in the experimental station for pigs at the Faculty of Agrobiology, Food and Natural Resources, Czech University of Life Sciences in Prague (Czech Republic). Immediately after arrival, they were individually weighed and tagged with ear tags and chips and then divided into three groups (each of 12 animals with equal gender representation): control (C) and two experimental groups (B-20 and B-60). Each group of pigs (control or experimental) was kept in a separate pen and had ad libitum access to feed and drinking water during the entire test period.

Before the actual test, pigs were subjected to an adaptive period lasting 5 days. During this period, all pigs were fed with same diet. It was based on barley, wheat, soybean meal, soy toasted beans, and maize, and supplemented with sodium chloride, calcium carbonate, and calcium dihydrogen phosphate. After the adaptive period, feeding commenced with doses of bisphenol A (BPA) and bisphenol AF (BPAF). All groups were provided with the feed mixture in self-feeders.

The control group continued to be fed without any addition of bisphenols. Group B-20 was fed a mixture of 20 µg BPA and BPAF (10 µg BPA + 10 µg BPAF)/kg b.w./day for 21 days. Group B-60 was administered the same mixture of BPA and BPAF, but at a higher concentration of 60 µg/kg (30 µg BPA + 30 µg BPAF) b.w./day for 21 days. These concentrations were converted to the average live weight of pigs and daily feed consumption to obtain the amounts needed to be mixed into the diet. The human reference dose of tolerable daily intake (TDI) for BPA is 50 µg/kg b.w./day, according to EPA (USA). Given the metabolic similarity between pigs and humans, we decided to use a higher concentration (60 µg). The use of a lower concentration (20 µg) is based on the knowledge that the actual exposure to BPA for humans is currently about 3–5 times lower than TDI. For the evaluation of growth intensity, the animals were divided into two groups based on their average daily gain (ADG): low ADG (below 435 g/day) and high ADG (above 435 g/day).

### 2.2. Slaughtering and Sampling

The slaughtering of pigs was performed according to the scheme illustrated in Table 1. On the second day after 21 days (T0) of BPA+BPAF administration, the entire control group and 4 pigs from B-20, as well as B-60, were slaughtered at a commercial abattoir under standard conditions. Blood samples were taken during exsanguination and then centrifuged to obtain the blood plasma. After cutting the carcasses, samples of liver, kidney, lung, spleen, muscle (*musculus longissimus lumborum et thoracis*), and fat tissue (back fat above the 7th vertebra) (each approximately 20 g) were taken. Immediately after collection, samples were transported within one hour to the laboratory at the Department of Animal Science, where they were frozen at −80 °C and stored until analysis. Both plasma and liver samples were taken and stored in glassware to avoid bisphenol contamination.

Fourteen (T14) and twenty-eight (T28) days, respectively, after finishing bisphenol administration, four pigs from both experimental groups, B-20 and/or B-60, were slaughtered, and samples were taken in the same manner as in the previous case.

### 2.3. Equipment

The equipment included a gas chromatograph, GC-2010 Plus (Shimadzu Corporation, Analytical Instruments Division, Kyoto, Japan), equipped with an AOC—20i autosampler (Shimadzu) and interfaced with a single quadrupole GC-MS-QP2010 Ultra (Shimadzu). GC separation was performed in 2D mode on two ovens and two columns. The first column was a Supelco SLB—5ms column (30 m × 0.32 mm × 0.1 µm film thickness), and the second column was a Supelco SPB—50 column (30 m × 0.32 mm × 0.25 µm film thickness). Helium (5.0) was used as a carrier gas with a constant flow of 2 mL/min.

### 2.4. GC-MS Analysis

BPA (99% purity), BPA-d16 (97.9% purity), and BPAF (99.9% purity) were purchased from Dr. Ehrenstorfer (LGC Limited, Vaughan, ON, Canada). Individual solutions of the standards were prepared in ethyl acetate (purity 99.9%, VWR International, Czech Republic), and the internal standard was prepared in methanol (purity 99.9%, Honeywell International, Praha, Czech Republic).

Acetonitrile for HPLC assay (99.95% purity) was purchased from VWR Chemicals (VWR International, Stříbrná Skalice, Czech Republic).

BSTFA—N, and O-Bis(trimethylsilyltrifluoroacetamide) with 1% trimethylchlorosilane were from Sigma-Aldrich Handels Gmbh (Wien, Austria).

The analysis was performed in two GC dimensions with the same temperature program. The initial column temperature of 130 °C was held for 1 min, followed by a linear increase at a rate of 20 °C per minute to 250 °C, held for 2 min, followed by a linear increase at a rate of 10 °C per minute to 280 °C, held at 280 °C for 11 min. The total program time was 23 min. After 6 min, the eluent from the first dimension was switched to the second dimension. Switching recovery was 100%, and the switching window was 6–23 min. The injector was set at 250 °C, the ion source at 230 °C, and the interface at 250 °C. The MS detector was set in selected ion monitoring (SIM) mode, detecting two ions per analyte (Table 1). A sample injection of 1 µL was performed in splitless mode. The analytical performance of the developed method is shown in Table 2.

### 2.5. Quality Control

Glassware was preferentially used to avoid potential BP contamination from the plastic material. At the beginning of the analytical procedure, samples were spiked with the internal standard (IS) BPA-d16. Linearity was assessed by multilevel calibration with five calibration levels. Calibration curves were built with the least squares linear regression model, plotting the peak area ratios of target compounds and IS versus their concentration ratios.

### 2.6. Sample Preparation

The samples were prepared according to Deceuninck et al. [24], with some modifications. To 1 g of tissue sample, 3 mL of acetonitrile and 100 µL internal standard were added, homogenized, and shaken for 30 min at room temperature. The sample was then centrifuged at 4000 rpm for 10 min at 4 °C to remove sediment. The supernatant was transferred to a new polypropylene tube and evaporated to dryness at 45 °C. The extract was reconstituted in 1 mL of water/methanol (90/10, *v*/*v*) and loaded into an SPE cartridge (Chromabond HLB, 60 µm/3 mL/60 mg, Machery-Nagel, Dueren, Germany), which had been preconditioned with 5 mL of methanol and 2.5 mL of deionized water. After sample loading, the SPE cartridge was washed with 3 mL of deionized water and then with 4 mL of a 90/10 (*v*/*v*) deionized water–methanol mixture. The analytes were eluted with 1 mL of methanol and then evaporated to dryness. Then, 100 µL methanol was added to the sediment and evaporated to dryness. The samples were subsequently derivatized with 100 µL BSTFA (N, O-Bis(trimethylsilyl) trifluoroacetamide) containing 1% trimethylchlorosilane, as described by Wang et al. [25]. The derivatized samples were analyzed by two-dimensional GC-MS with parameters listed in Table 2 and Table 3.

### 2.7. Statistical Analysis

Association analysis was calculated using the generalized linear model (GLM) in SAS (Statistical Analysis System, Inst. Version 9.4, SAS Institute, Cary, NC, USA). The outcome variables of BPA and BPAF content in each tissue were analyzed with two-way analysis of variance (bisphenol administration—control, B20, and B60; time from the end of administration to slaughter—T0, T14, and T28), and calculations were performed for each time of administration (T0, T14, T28). The resulting data are presented in tables as LS means. Differences were considered significant at *p* < 0.05 and *p* < 0.01.

## 3. Results

In piglets slaughtered immediately after finishing the administration of bisphenols (T0), concentrations of BPA in plasma and organs were the highest in group B-60, except for lungs and kidneys (Table 4). Differences in plasma between the control and B-60 group were significant (*p* < 0.01). Additionally, significant differences were found between the two experimental groups for both muscle and plasma (*p* < 0.05). For BPAF concentrations, significant differences between control and both experimental groups were found (*p* < 0.01) (Table 5).

Concentrations of BPA two weeks after the administration of bisphenol are shown in Table 4. In most traits, higher values were found in group B-60 than in B-20, but the differences between them were not significant. Regarding bisphenol AF, concentration was found only in plasma, but differences were not significant (Table 5).

Similarly to the previous case, four weeks after finishing bisphenol administration, bisphenol concentrations in plasma and tissues were not significantly different between the control and experimental groups. On the other hand, concentrations were mostly higher in B-60 than in B-20. Regarding BPAF concentrations, they were found only in the B-60 group (5).

To monitor the accumulation and elimination of bisphenols from individual tissues, this study examined differences in BPA and BPAF concentrations in the plasma and tissues of pigs within the experimental groups (B-20/B-60), depending on the time elapsed between the end of bisphenol administration and slaughter (T0, T14, and T28) (Table 4 and Table 5).

In pigs exposed to lower dose (B-20), significant differences were found for BPA in liver between T14 and T28, as well as in muscle and fat between T0 and T28 (*p* < 0.05). Table 4 and Table 5 show a gradual decrease in the plasma BPA and BPAF concentrations with increasing time between the end of bisphenol administration and slaughter: a phenomenon not previously observed in any other tissue. On the contrary, an increasing tendency for BPA concentration was observed in the lungs, muscle tissue, spleen, and fat. The liver (highest BPA concentration at T14) and kidney (highest BPA concentration at T28, lowest at T14) do not exhibit such clear tendencies, suggesting that BPA accumulation does not occur in all tissues at the same rate, i.e., with the same intensity. Therefore, depending on the time elapsed since the end of BPA administration, it cannot be reliably stated whether the concentration will decrease or increase.

The same comparison was performed for group B-60. The only tissue that showed a significant difference was muscle, specifically between T14 and T28 (*p* < 0.05). However, there was a gradual increase in BPA concentration, depending on the time of slaughter, in only one tissue—the lungs —which indicates that the trend differs depending on the dose of BPA administered to the animals.

In addition to investigating the effect of bisphenol administration on their concentration in the various organs of pigs, our study also focused on the effect of growth intensity (ADG) on the content of BP and BPAF in pig bodies. The average daily gain for the entire group was 435 ± 69 g per day. With higher levels of bisphenols administered in the feed, a decrease in ADG was observed (Table 6), although this difference was not statistically significant.

These relationships are shown in the B-20 and B-60 groups, respectively (Table 7 and Table 8). The differences found between the low (below 435 g/day) and high daily gain (over 435 g/day) groups were not statistically significant, indicating that growth rate does not affect the concentration of bisphenols in the above tissues. Despite the above fact, a slightly increased concentration of bisphenols was found in most organs within both groups of pigs with a higher average daily gain (ADG). However, in some cases, higher concentrations of bisphenols were found in pigs with lower ADG, specifically in lungs for both the B-20 and B-60 groups, in plasma for B-20, and in fat for B-60.

## 4. Discussion

To the best of the authors’ knowledge, there have been no other studies concerning the concentrations of BPA and/or BPAF in pig plasma and body tissues collected immediately after finishing bisphenol administration, as well as two or four weeks after that. Only one study has described the level of BPA in the pig muscle after oral administration [26]. Therefore, comparing the results was quite challenging, and in several cases, we considered the results from human studies.

The presented results clearly demonstrate the occurrence of BPA and BPAF in the plasma and organs of pigs not only immediately after bisphenol administration but also two and/or four weeks after that. In our case, this is a pilot study conducted on 36 pigs (four animals per group), which may limit the statistical significance of the observed results. We recommend further experiments with a larger number of animals to more accurately monitor the accumulation and elimination of bisphenols.

Although polluted air and water certainly contribute to the accumulation of these components in animal tissues, we suppose that the main source of contamination in our experiment was the feed. Food is also the main source of bisphenols in humans [3,27]. Moreover, current automated feeding systems utilize feed hoppers and conveyors made of plastic materials. In addition to primary contamination with bisphenols in the production of feed mixtures, there can be secondary contamination, which is caused by the rolling of the inner plastic parts from the hoppers and conveyors during the transport of the feed to the barn. In addition, pigs stand on perforated plastic floors and receive feed from partially plastic automatic feeders, which increase contact with possible bisphenol contamination. It seems that all or part of these plastic elements may contain higher levels of bisphenols. At this point, it may be mentioned that there are no prohibitions or restrictions on the use of products containing BPA or their analogues in farm animals, unlike those intended for humans.

Moreover, it cannot be excluded that at least part of the BPA/BPAF contamination could have come from the mother’s body, both by sucking breast milk [28] and by transfer through the placenta [29], as the pigs in our experiment were very young.

The fact that BPA and BPAF were also analyzed in plasma and tissue samples from the control group suggests environmental pollution in the area where pigs are kept and fed. They can be exposed to both BPA and BPAF from polluted air, water, and feed. Despite the fact that research on bisphenol content in pig feeds has not been carried out so far, several studies on food for other farm or pet animals [30,31] or in environmental matrices [32] have shown the presence of these substances. Therefore, it can be assumed that BPA or BPAF are also present in the feed mixtures or in drinking water for pigs. Another possible source of the contamination of pigs with bisphenols may be transdermal transport [33] or the digestive tract (through biting) when pigs come into contact with plastic materials.

Concentrations of BPA and BPAF were highest in the group that was fed a higher daily dose of bisphenols (60 µg), followed by the lower dose (20 µg) and the control group. The presented results showed that concentrations of BPA in pigs’ plasma were higher than those of BPAF, and they both decreased with increasing time in both experimental groups. This is most likely due to the fact that bisphenol A/AF moves from the blood to the liver and, consequently, to other organs.

Accumulation in the liver is generally dynamic because metabolic processes actively process and transform these substances into excretable products. When the concentration of these compounds increases, the liver enhances its activity to break down and eliminate them from the body. BPA and BPAF are metabolized specifically in the liver, where phase I and phase II enzymes modify the molecules of these toxins into less harmful forms that are subsequently excreted from the organism [34,35,36].

A decrease in concentration at T28 was also observed in the liver, primarily because the liver is the primary site of BPA metabolism. As it is known, BPA in humans and some primates is rapidly conjugated in the liver with glucuronic acid to the inactive BPA-glucuronide. A lesser amount of BPA might also be sulfated to form BPA-sulfate. These conjugates are transported to the blood to reach the kidneys, where they are further excreted in the urine. Some small amount (less than 1%) of unconjugated BPA circulates in the blood and might be excreted in feces or accumulated in tissue later. Since pigs are metabolically close to humans, it can be assumed that the metabolism of BPA in pigs’ bodies is similar to that of humans. The fact that BPA is excreted together with urine could explain why a relatively high concentration was detected in the kidneys and also why it had a relatively increasing tendency. This phenomenon is confirmed, for example, by Murakami et al. [37], who state that if more BPA enters the kidneys than can be excreted in the urine, it accumulates in the kidney tissue. The schema for the metabolism of BPAF is similar, but there are some differences. BPAF is also metabolized in the liver, to a lesser extent in the intestines [38], and to the glucuronides and/or sulfates. Some studies on rodents suggest that BPAF is excreted mainly through the feces 72 h after oral administration, and, to a lesser extent, via urine [39].

More interesting is the opposite tendency in some tissues: namely, the tendency for BPA concentration to increase over time. An explanation for muscle tissue can be found in Makowska et al. [26], who investigated the accumulation of BPA in muscles when it is ingested “per os”. They state that with the intake of higher doses of BPA, its accumulation occurs in the muscles due to the body’s ability to degrade only a certain amount; if BPA is ingested “above the limit”, it enters other tissues through the blood.

Similarly to muscle tissue, the concentration of BPA was also found in the spleen, with an increasing tendency observed in group B-20. In group B-60, the concentration of BPA in this organ reached its highest values at T14. This suggests that spleen tissue is highly sensitive to bisphenol A, and BPA is likely to accumulate there.

In the lungs, an increase in concentration was also recorded over time, rather than a decrease, which could be caused by BPA being absorbed into the body even after administration via the respiratory route, a phenomenon that cannot be confirmed or refuted. Another possibility for explaining the increase could be the gradual accumulation of BPA in the lungs, where lipophilic substances can accumulate primarily due to the lipid content in the lung surfactant. The effect of BPA on rat lung tissue was studied by Yoo et al. [40]. BPA was also administered to them orally, and, according to the research results, it caused relatively serious changes in the structure of the lung tissue. These changes then led to the development of pulmonary fibrosis. Based on this source, it can be confirmed that when taken orally, bisphenol A is absorbed into the bloodstream and subsequently enters the lungs.

The last organ where BPA concentration increased over time was adipose tissue, or fat. This phenomenon can be explained by the similarity to the lungs, i.e., the affinity of BPA to adipose tissue and fat cells. Therefore, it is understandable that accumulation occurs in this area. At the same time, BPA is gradually released from the fat, which explains the varying values in other organs. This phenomenon is mentioned by Berni et al. [41], who also note that the concentration of BPA in fat tends to be the lowest compared to other types of tissue, which is a peculiarity that is difficult to explain.

Regarding the relationship between the accumulation of BPA/BPAF in the organs of pigs and growth rate, it was not possible to demonstrate that growth intensity had a statistically significant effect on the concentration of BPA in the analyzed tissues. However, there was a certain tendency to retain more BPA in the body of piglets with higher daily gain, especially in group B-60. In this group, animals with higher ADG accumulated more BPA in all tissues, except for lungs and fat, than pigs with lower ADG. In group B-20, a higher growth rate was associated with higher BPA concentrations in the liver, muscle, spleen, fat, and kidney, while a lower ADG was associated with higher BPA concentrations in plasma and lungs.

Findings of our study suggest that oral exposure of pigs to BPA/BPAF might be reflected in the level of these substances in the organs of animals, and therefore, eating those parts may contribute to higher exposure to these bisphenols in humans. Future studies should focus not only on the targeted administration of known bisphenol concentrations in feed, but also on analyzing potential sources of bisphenol contamination, such as the environment, feed, water, and stable conditions.

## 5. Conclusions

The results of the present study have shown that oral administration of bisphenols led to an increase in the concentration of BPA and BPAF in plasma and other tissues of young pigs. These higher concentrations were determined by slaughtering the animals immediately after finishing the oral exposure, as well as two and four weeks later. The incidence of both bisphenols in the control pigs also suggests contamination of the farm environment with these substances. This may represent a potential risk not only for animal health but also for human health, through the consumption of pig organs. From the perspective of food safety and animal welfare, these findings indicate the need for further investigation into possible sources of bisphenol contamination on pig farms and ways to minimize BPA/BPAF levels in materials used in animal production and packaging. These results represent a pilot study of our research that focused on the effect of oral administration of two bisphenols on their occurrence in various pig tissues. Despite its limitations, this pilot study may contribute to a better understanding of food safety issues related to bisphenols in animal farming and feed production. 

## Figures and Tables

**Table 1:** Slaughter scheme of pigs.

Group/Time	T0 ^1^	T14 ^1^	T28 ^1^	Σ
Control (n)	12	-	-	12
B-20 (n)	4	4	4	12
B-60 (n)	4	4	4	12
Σ	20	8	8	36

^1^ Time of slaughtering at the following: T0—immediately after bisphenols administration, T14—two weeks after bisphenols administration, and T28—four weeks after bisphenols administration.

**Table 2:** GC-MS acquisition parameters.

Analyte	RT (min)	Ions (*m*/*z*)	
		Quantitative	Qualitative
BPAF	10.97	411	480
BPA-d16	13.38	368	369
BPA	13.48	357	358

**Table 3:** Analytical performance of the developed method.

Compounds (µg/L)	Coefficient of Determination (R^2^)	LOD (µg/L)	LOQ
BPAF	0.999	0.60	1.99
BPA	0.999	0.88	2.94

**Table 4:** Concentration of BPA (ng/g) in pigs’ tissue.

Trait	Time	Control	B-20	B-60
Plasma	T0	5.73 ± 6.40 ᴬ	23.12 ± 7.52 ᵃ	55.07 ± 7.52 ᴮᵇ
	T14		15.85 ± 8.23	27.35 ± 7.13
	T28		12.19 ±7.69	23.84 ± 7.69
Liver	T0	7.16 ± 2.10	8.04 ± 3.55	10.78 ± 4.10
	T14		10.91 ± 4.39	9.25 ± 4.39
	T28		2.18 ± 0.62	3.20 ± 0.62
Lungs	T0	8.68 ± 3.06	7.37 ± 1.86	5.76 ± 1.86
	T14		10.08 ± 10.46	12.70 ± 8.54
	T28		16.96 ± 9.12	16.98 ± 7.90
Muscle	T0	9.55 ± 6.55 ^ab^	5.80 ± 3.32 ^a^	16.23 ± 3.32 ^b^
	T14		22.71 ± 16.16	33.09 ± 16.16
	T28		25.22 ± 6.41	8.86 ± 6.41
Spleen	T0	14.34 ± 4.41 ᵃ	19.82 ± 7.37	29.04 ± 6.38
	T14		20.81 ± 5.57	18.38 ± 6.83
	T28		33.46 ± 9.93	25.97 ± 8.60
Fat	T0	0.57 ± 0.19	0.47 ± 0.27	0.69 ± 0.27
	T14		1.12 ± 0.26	1.31 ± 0.26
	T28		1.65 ± 0.31	1.17 ± 0.31
Kidney	T0	16.96 ± 3.10	23.08 ± 5.90	16.96 ± 3.10
	T14		16.47 ± 5.29	29.84 ± 5.29
	T28		27.47 ± 5.40	24.16 ± 4.67

^a,b^ *p* < 0.05, ^A,B^ *p* < 0.01.

**Table 5:** Concentration of BPAF (ng/g) in pigs’ tissue.

Trait	Time	Control	B-20	B-60
Plasma	T0	0.17 ± 0.42 ^A^	10.28 ± 2.85 ^B^	9.48 ± 3.74 ^B^
	T14		6.80 ± 3.74	4.55 ± 2.85
	T28		n.d.	0.94 ± 0.51
Liver	T0	0.12 ± 0.16	0.25 ± 0.20	n.d.
	T14		n.d.	n.d.
	T28		n.d.	0.71 ± 0.50

^A,B^ *p* < 0.01. Note: Concentrations of BPAF in other tissues were not detectable (n.d).

**Table 6:** Average daily gain (g/day).

Trait	Control	B-20	B-60
ADG	455 ± 67	450 ± 61	421 ± 88

**Table 7:** Effect of ADG (g/day) on BPA (ng/g) concentrations in plasma and tissues of pigs exposed to a lower dose (B-20).

Trait	Low ADG (Below 435/g Day)	High ADG (Over 435 g/Day)
ADG	376 ± 30	486 ± 29
Plasma	13.64 ± 7.98	18.48 ± 9.61
Liver	2.73 ± 1.41	9.20 ± 7.7
Lungs	16.96 ± 18.94	8.28 ± 3.90
Muscle	30.83 ± 23.51	11.45 ± 13.76
Spleen	30.94 ± 25.52	21.57 ± 7.94
Fat	1.26 ± 0.54	0.99 ± 0.79
Kidney	25.15 ± 15.37	20.94 ± 3.68

**Table 8:** Effect of ADG (g/day) on BPA (ng/g) concentrations in plasma and tissues of pigs exposed to a lower dose (B-60).

Trait	Low ADG (Below 435/g Day)	High ADG (Over 435 g/Day)
ADG	352 ± 55	490 ± 52
Plasma	34.71 ± 18.42	36.13 ± 31.7
Liver	2.95 ± 1.56	12.89 ± 11.87
Lungs	7.52 ± 4.18	16.78 ± 17.02
Muscle	5.19 ± 4.91	33.60 ± 25.36
Spleen	36.26 ± 19.61	18.62 ± 8.73
Fat	1.31 ± 0.44	0.81 ± 0.80
Kidney	29.47 ± 8.56	18.99 ± 4.91

## Data Availability

None of the data were deposited in an official repository. The data that support the study findings are available to reviewers.
